# Analysis of Formation Mechanisms of Sugar-Derived Dense Carbons via Hydrogel Carbonization Method

**DOI:** 10.3390/nano12224090

**Published:** 2022-11-21

**Authors:** Liting Chen, Zheqiong Fan, Weiguo Mao, Cuiying Dai, Daming Chen, Xinghong Zhang

**Affiliations:** 1 School of Materials Science and Engineering, Xiangtan University, Xiangtan 411105, China; 2 College of Materials Science and Engineering, Changsha University of Science and Technology, Changsha 410114, China; 3 National Key Laboratory of Science and Technology on Advanced Composites in Special Environments, Harbin Institute of Technology, Harbin 150000, China

**Keywords:** sugar, polyacrylamide, hydrogel, carbon

## Abstract

Four kinds of sugar (glucose, fructose, sucrose, and maltose) were selected as carbon precursors, and corresponding dense carbon products were prepared using a novel hydrogel carbonization method. The carbonization processes of sugar–polyacrylamide (sugar–PAM) hydrogels were studied in detail. The molecular structures in the raw materials were analyzed by proton nuclear magnetic resonance spectroscopy (^1^H NMR). Samples prepared at different temperatures were characterized by thermogravimetry analysis (TGA) and Fourier-transform infrared (FTIR) spectroscopy. The morphology and microstructure of sugar-derived carbons were confirmed by field-emission scanning electron microscopy (FESEM) and X-ray diffraction (XRD). The results indicated that the sugar solution was surrounded by PAM with a three-dimensional network structure and formed hydrogels in the initial stage. The sugar solution was considered to be separated into nanocapsules. In each nanocapsule, sugar molecules could be limited within the hydrogel via walls formed by PAM chains. The hydroxyl group in the sugar molecules connected with PAM by the hydrogen bond and intermolecular force, which can strengthen the entire hydrogel system. The self-generated pressure of hydrogel constrains the foam of sugar during the heat treatment. Finally, dense carbon materials with low graphitization instead of porous structure were prepared at 1200 °C.

## 1. Introduction

Nowadays, carbon materials are widely used in many fields because of their diverse properties and structures. Along with the rapid growth in carbon materials, however, there is increasing concern over the precursors of these materials. Traditionally, various hydrocarbons [1,2,3,4], pitches [5,6,7], and resins [8,9] are the main sources of carbon materials, which are nonrenewable resources. A large number of energies are needed, and substantial quantities of pollutants are produced during the manufacturing processes. As a result, the sustainable carbon producing from readily available and renewable resources, especially biomass, have been attracting attention due to the emergence of the energy crisis and environmental pollution. As a promising representative of biomass, sugars are of interest due to their extensive sources and simple compositions (C, H, O) as carbon precursors, indicating low preparation cost and pollutant emission.

In the last few decades, various carbon materials produced from different sugars, such as glucose [10,11], fructose [12,13], sucrose [14,15], and maltose [16,17], have been reported. Sugars have been used to fabricate porous carbons using direct pyrolysis. A great quantity of gases, such as H_2_O, CO_2_, CO, CH_4_, form and release from the parent phase during thermal treatments, resulting in the expansion and foaming of naturally organized structures of sugar [18]. Huang et al. found that hard carbon microspheres can be prepared from sugar by a hydrothermal method [19]. Subsequent works reported the formation process of carbon microspheres in detail, including the hydrothermal mechanisms, product chemistry, and structural characterization. In addition, carbon quantum dots [20] and graphene foams [21] have also been produced by utilizing sugar sources.

Porous and low-dimensional carbon materials derived from sugar have become the main carbon products. However, the low yield and complex preparation processes limit their applications. Sun et al. recently proposed a new simple hydrogel method to prepare bulk dense carbon materials with high residual carbon ratio from sugar [22]. They introduced sugar into a polyacrylamide (PAM) gel system and formed dense isotropic nanoscale polycrystalline graphite through critical thermal treatment. By controlling microstructure, crystal orientation, and bonding mode of sugar-derived carbon, it is possible to produce structurally and functionally integrated carbon materials with various excellent properties. Based on the convenient hydrogel method, different kinds of carbon materials have been fabricated. Tan et al. prepared a glucose-derived anisotropic carbon film with high in-plane thermal conductivity and outstanding electromagnetic interference using this method [23]. The carbon retention rate of sugar is higher than 82%. Carbon fiber was also prepared by glucose and sucrose by Yang et al. [24]. The as-prepared carbon fibers have dense and homogeneous microstructure and excellent mechanical property. Multifunctional sucrose-derived carbon foams with excellent heat insulation and flame retardancy were fabricated by a template-free mechanical foaming gel method [25]. These interesting works provide a new insight into the manufacture of carbon materials from sugar. However, the formation mechanisms of these sugar-derived biocarbons have been less studied. More research is required to reveal the effect of hydrogel on final carbon performances and chemical transformations during thermal treatment.

In this work, four sugars—glucose, fructose, sucrose, and maltose—were selected as carbon precursors and prepared to internal dense bulk carbon by the hydrogel carbonization method, respectively. The cross-linking behavior of sugar and acrylamide (AM) were evaluated by proton nuclear magnetic resonance spectroscopy (^1^H NMR). The structural change in hydrogel materials under different temperatures was characterized by thermogravimetry analysis (TGA) and Fourier-transform infrared (FTIR spectroscopy). The microstructure and components of the bulk carbon materials were analyzed by X-ray diffraction (XRD) and field emission scanning electron microscope (FESEM). The formation mechanisms of the four sugar-derived carbon materials in the presence of hydrogel are systematically discussed and the effect of different sugar sources on the final product analyzed. The results will be useful in better understanding the carbonization mechanisms of sugar–hydrogel systems.

## 2. Materials and Methods

### 2.1. Materials

The glucose, fructose, and sucrose were purchased from Tianjin Kermel Chemical Reagent Co., Ltd. (Tianjin, China). The maltose was purchased from Shanghai Aipi Chemical Reagent Co., Ltd. (Shanghai, China). The monomer, AM, and the cross-linking agent, N,N’-methylenebisacrylamide (MBA), were supplied by Shanghai Aladdin Biochemical Technology Co., Ltd. (Shanghai, China). Selected as an initiator, 2,2′-Azobis(2-methylpropionamidine) dihydrochloride (AIBA) was provided by Shandong Xinheng Chemical Reagent Co., Ltd. (Jining, China).

### 2.2. Preparation of Sugar–Polyacrylamide

**Figure 1:**
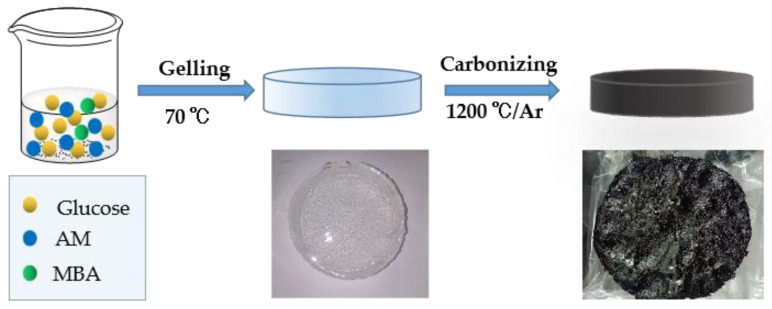
Process of sugar-derived carbon via hydrogel.

The preparation steps of hydrogel carbonization were as follows. Firstly, 200 g glucose was dissolved into 100 g deionized water in a 500 mL beaker. Then, AM (12 g) and MBA (0.48 g) were successively added into the beaker. Lastly, 1.6 g 10 wt.% AIBA was added as the initiator for the polymerization of AM. After all the materials had dissolved completely, the mixture solution was placed in a drying oven at 70 °C to form transparent and monolithic glucose-containing polyacrylamide hydrogel (Glu–PAM hydrogel). To make the carbonization process gentler, precarbonization was adopted. Later, the as-prepared hydrogel was dried at 100 °C for 24 h, 160 °C for 10 h, 200 °C for 10 h, and 240 °C for 10 h in turn to obtain a precarbonized solid sample. Lastly, the sample was carbonized at 1200 °C at 5 °C/min heating rate in an argon atmosphere. The other three sugars (fructose, sucrose and maltose) were treated via the same processes with glucose, and the obtained hydrogels were named Fru–PAM, Suc–PAM and Mal–PAM, respectively. The process of preparation can be seen in Figure 1.

### 2.3. Characterization Methods

Glucose and AM were dissolved in D_2_O and the temperature kept at 70 °C to form the sample G. The samples F, S, M were obtained by using fructose, sucrose and maltose as raw material instead of glucose. The four kinds of sugar—AM and sample G (F, S, and M)—were analyzed by nuclear magnetic resonance imaging (Bruker AV400, Billerica, MA, USA) to determine if a chemical reaction between AM and sugar had occurred. Since hydrogel contains abundant oxygen-containing functional groups that lead to hydrogel good hydrophilicity, the sugar–PAM hydrogel was heat-treated at 100 °C for 24 h to eliminate the effect of free water and then investigated by TGA with a TA Instrument analyzer (TGA 50, Kyoto, Kyoto-fu, Japan) at a heating rate of 6 °C/min from ambient temperature to 950 °C in N_2_ atmosphere. In addition, Fourier-transform infrared spectroscopy (Nicolet 380) was used to analyze the variations in functional group of samples after different temperature treatments. The FTIR samples were prepared using the KBr pellet technique. The microstructures and morphologies of the final four carbonized samples were characterized by XRD (Rigaku Ultima IV, Tokyo, Kyoto-fu, Japan) and FESEM (TESCAN MIRA3 LMU, Brno, South Moravia, Czechia). The scanned angles ranged from 0° to 90° with a step size of 8° using an X-ray diffractometer equipped with Cu Ka radiation (λ = 0.154 nm).

## 3. Results

**Figure 2:**
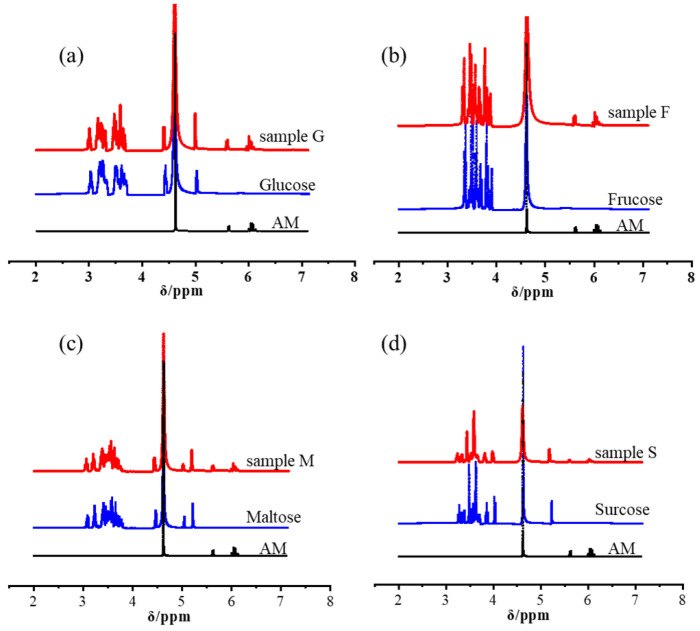
The ^1^H NMR spectra of (**a**) glucose, AM and sample G, (**b**) fructose, AM and sample F, (**c**) maltose, AM and sample M, (**d**) sucrose, AM and sample S.

Figure 2 shows the ^1^H NMR spectra of sugars, AM, and the sample G (F, M, S). As can be seen, there was no new chemical bond in sample G (F, M, S), indicating that none of the four sugars reacted with AM. Therefore, sugars were fixed in a three-dimensional network of polyacrylamide by hydrogen bonds and intermolecular forces during the formation of sugar–PAM hydrogel. The results were consistent with Dutta’s research on chitosan and PAM hydrogel materials, in which stiff and tough supramolecular polymer hydrogels can also be formed without the presence of covalent bonds [26].

**Figure 3:**
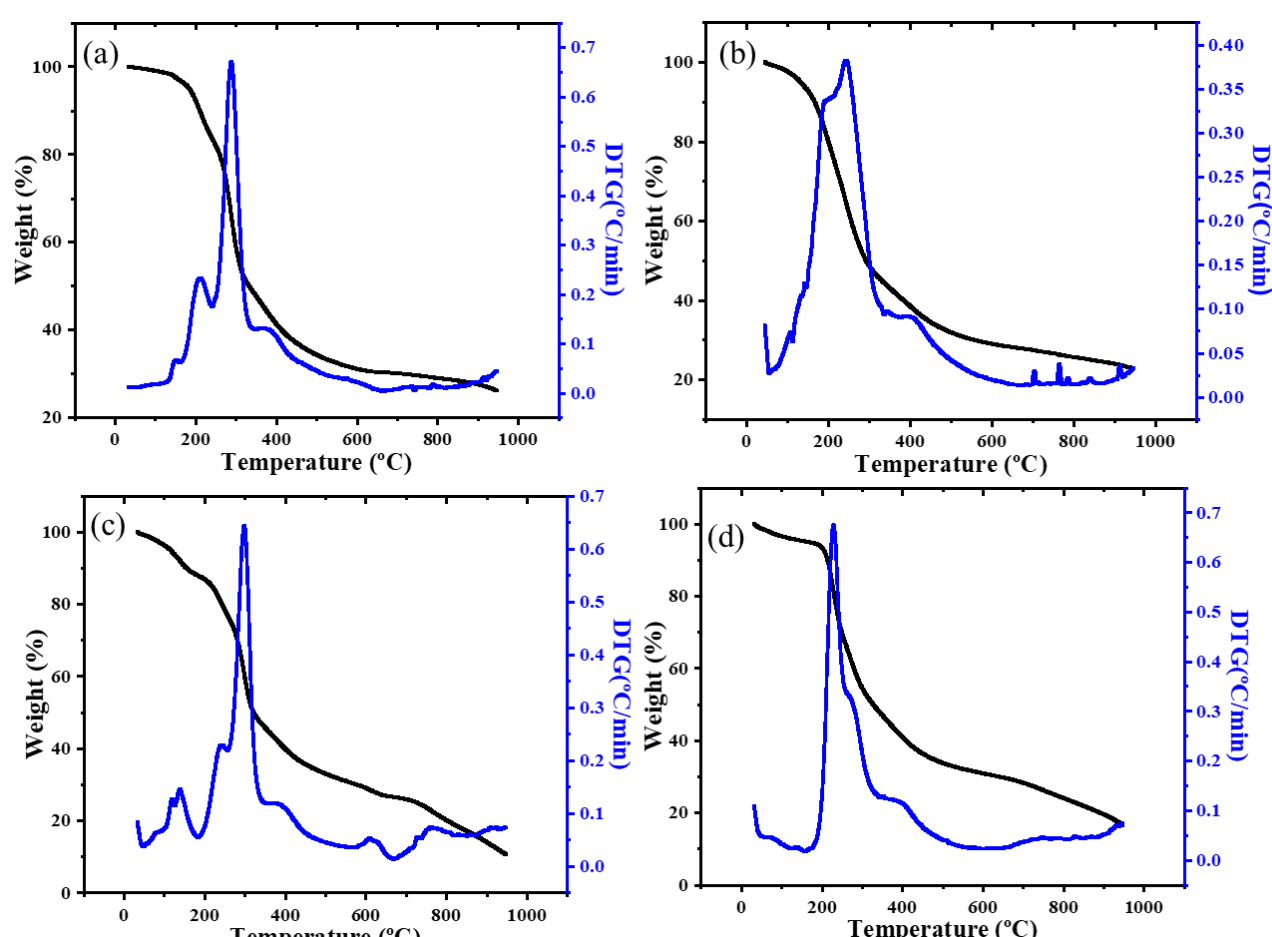
TGA and DTG curves of four sugar–PAM hydrogels (**a**) Glu–PAM, (**b**) Fru–PAM, (**c**) Mal–PAM hydrogel, (**d**) Suc–PAM hydrogel.

The thermal decomposition results of the four sugar–PAM samples are given in Figure 3 and the peak temperature of four sugar–PAM in the DTG curve are given in Table 1. As shown in Figure 3a, two obvious peaks located at 208.9 °C and 288.1 °C can be observed with corresponding weight loss of 10.37% and 33.59%, respectively. Compared with the original glucose’s DTG, the number of peaks in pure glucose and Glu–PAM samples are the same, but there is a difference in peak positions. The pure glucose has two peaks located at 215 °C and 275 °C [27]. According to F.ORS’s research, intermolecular dehydration reactions occur between parts of glucose within 240 °C, resulting in the formation of oligomers and water [27]. Hence, the weight-loss peak at 208.9 °C should result from the evaporation of water comprising the bound water in Glu–PAM hydrogel and reaction product water in the dehydration process. With the increase in temperature, the formed oligomers undergo decomposition and cyclization reactions successively, resulting in the formation of a second weight-loss peak at 288.1 °C. In Figure 3b, the DTG curve of Fru–PAM has only one peak at 242.1 °C with about 34.84% weight loss. Similarly, the DTG curve of pure fructose also has only one peak at 205 °C [27], also caused by the intermolecular dehydration reaction. The lower reaction temperature means that fructose is more prone to intermolecular condensation than glucose [28]. Figure 3c,d show the DTG curves of Mal–PAM and Suc–PAM, the raw sugar materials, both of which are disaccharides. Maltose is formed by the combination of two units of glucose. Sucrose is composed of one unit of glucose and one unit of fructose. For Mal–PAM, three peaks are located at 139.4, 242.9 and 298.5 °C (see Figure 3c). For Suc–PAM, only one peak at 229.1 °C can be seen in Figure 3d. Based on the molecular structure information, both glucose units in maltose and glucose and fructose units in sucrose are linked by glycosidic bonds that can be easily broken by temperature activation [29]. Therefore, maltose and sucrose have similar pyrolysis behavior, including glycosidic bond cleavage, intermolecular dehydration condensation, and subsequent aromatization to form a carbon structure [30].

In general, the DTG curves of these sugar–PAM samples are similar to that of pure sugar raw materials [31,32,33]. The considerable shift in peak positions implies that the addition of PAM in sugar aqueous solution has important influence on the sugar’s pyrolysis behavior. In earlier studies, the Maillard reaction was considered to occur between sugar and PAM [24] in a temperature range of 90–130 °C [34]. Only after the rupture of glycosidic bond could the reducing sugar be formed to react with the amide group in the PAM. However, it should be noted that both the decomposition of sucrose–PAM and the break of the glycoside bond in sucrose occurred almost simultaneously at the single DTG peak of 229 °C, indicating that the Maillard reaction did not happen here.

**Figure 4:**
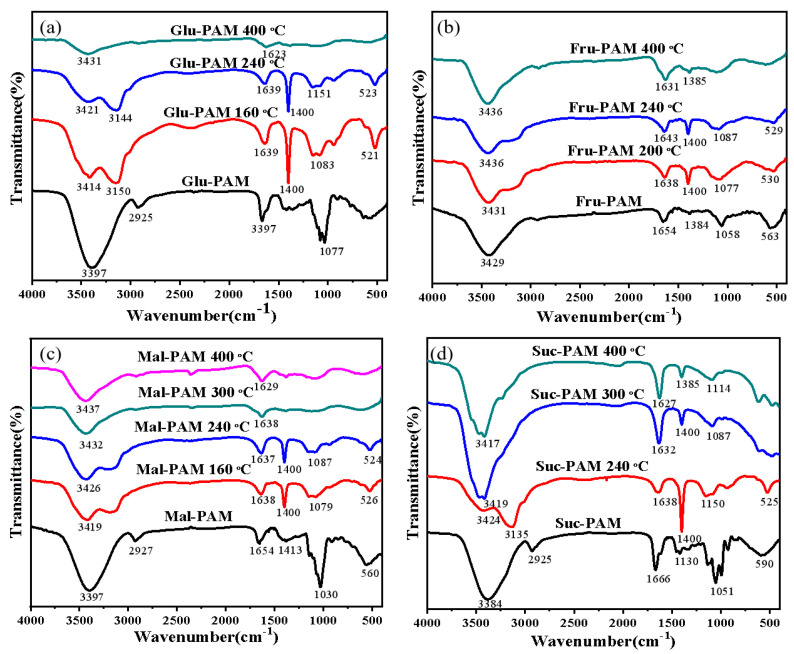
FTIR spectra of four sugar–PAM hydrogels treated at different temperatures. (**a**) Glu−PAM, (**b**) Fru−PAM, (**c**) Mal−PAM, (**d**) Suc−PAM.

In addition, FTIR was adopted to explore the possible reactions occurred in sugar–PAM samples during the pyrolysis process, and the results are presented in Figure 4. Based on the above DTG results, it can be seen that the pyrolytic behaviors of different sugar–PAM samples were rather different and related to the type of sugar source. Hence, the sugar–PAM samples heat-treated at various temperatures were analyzed. For example, the original Glu–PAM hydrogel treated at 160 °C, 240 °C, and 400 °C was chosen as the FTIR test sample. The broad band between 3000 and 3700 cm^−1^ stemmed from the O-H stretching vibration of water and sugar. The band at 3425 cm^−1^ represented the N-H stretching vibration of PAM [35], and the band at 2800–3000 cm^−1^ corresponded to stretching vibration of aliphatic C-H [36]. The presence of an aromatic ring was evidenced by the band at 1620–1680 cm^−1^ attribute to C=C and C=O vibrations, and the shared bands at 1000–1400 cm^−1^ indicated the coexistence of O=C-OH (carboxyl), C-O-C (epoxy) and C-OH (hydroxyl) [37]. In addition, the bands in the 750–875 cm^−1^ region were assigned to aromatic C-H out-of-plane bending vibrations [38].

As shown in Figure 4a, for the original Glu–PAM hydrogel, the broad absorption peak between 3000 and 3700 cm^−1^ were mainly caused by large amounts of solvent water. With the temperature increased, the water began to evaporate gradually, resulting in the presence of the hydroxyl absorption peak at 3151 cm^−1^ and N-H absorption peak at 3425 cm^−1^. The shared band at 1000–1400 cm^−1^ firstly appeared in Glu–PAM–160 °C, suggesting that dehydration reaction and formation of oligomer had taken place at 160 °C. Comparing Glu–PAM–160 °C with Glu–PAM–240 °C, there was little change in the curve shape, but great variation in the peak intensity. The biggest difference occurred at 1400 cm^−1^ and the intensity of peak decreased as increasing in temperature, and the peak nearly disappeared when the temperature rose to 400 °C (see Glu–PAM–400 °C). This change means that the amount of oligomer was reduced due to the aromatization reactions between themselves. From Figure 4b–d, the FTIR curves of the other three sugar–PAM samples (Fru–PAM, Mal–PAM, Suc–PAM) had developmental trends similar to Glu–PAM; therefore, no additional analysis was done here. Combining the FTIR results, it can be speculated that caramelization is the main reaction during the sugar–PAM pyrolysis process, which is consistent with the above DTG results.

Firstly, levoglucosan and pyranose is produced by part of glucose in Glu–PAM or part of maltose in Mal–PAM through intermolecular dehydration condensation [29], which can be evidenced by the enhanced intensity of C-O-C group absorption peak at 1400 cm^−1^ when the temperature increased to 160 °C (Figure 4a,c). Secondly, the intermediate products began to aromatize and form aromatic clusters, which can be identified from Figure 4a,c, with the peak intensity of C-O-C group reduced, while that of the corresponding C=O group at 1620–1680 cm^−1^ became stronger [10].

For Fur–PAM, fructose was firstly decomposed to form 5-(hydroxymethyl)-2-furaldehyde (HMF) [27,28,39], which can be demonstrated by the equivalent strength of the two absorption peaks at 1400 cm^−1^ and 1620–1680 cm^−1^, respectively. Subsequently, part of HMF was broken down to levulinic acid and formic acid via hydrolysis, which in turn accelerated the polymerization of HMF to produce continuous long molecular chains of carbon via intermolecular dehydration condensation [40]. These long chains would eventually constitute a carbon skeleton with spatial structure.

Unlike fructose, both HMF and glucose were generated by fracture of glycosidic bond of sucrose during pyrolysis [41]. As the temperature rose, intermolecular dehydration condensation caused the intensity of C-O-C functional groups’ absorption peaks to decrease, while that of C=O and C=C groups’ absorption peaks increased. Finally, the intensity of all the functional group decreases with the increasing of temperature, suggesting carbon was formed.

**Figure 5:**
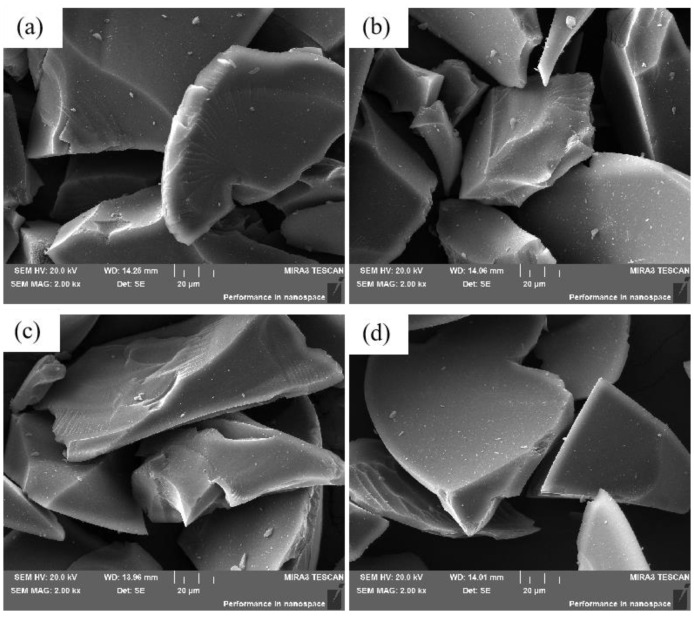
SEM images of different sugar-derived carbons carbonized at 1200 °C. (**a**) Glu−PAM, (**b**) Fru−PAM, (**c**) Mal−PAM, (**d**) Suc−PAM.

**Figure 6:**
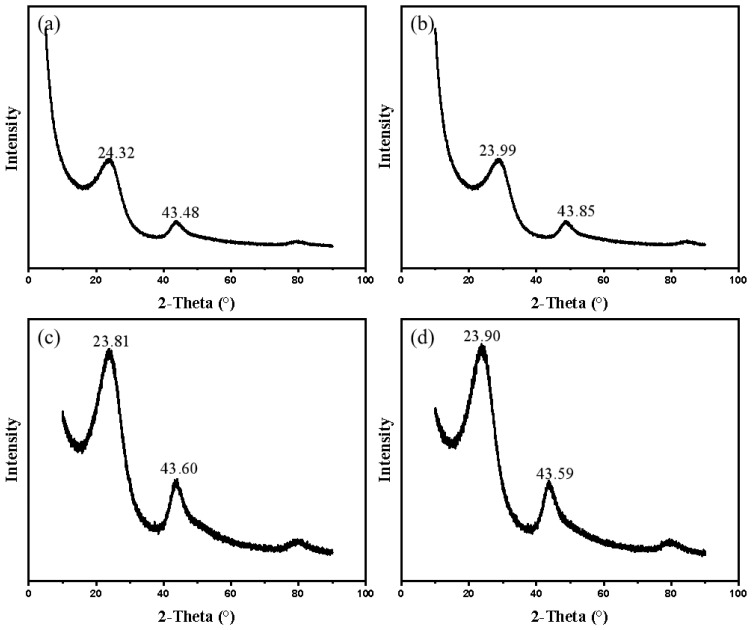
XRD patterns of different sugar-derived carbons prepared at 1200 °C. (**a**) Glu−PAM. (**b**) Fru−PAM. (**c**) Mal−PAM. (**d**) Suc−PAM.

The sugar–PAM hydrogel carbonization at 1200 °C appeared dense in structure [22,23], which indicated the sugar–PAM carbonizes completely at 1200 °C. Therefore, the morphology and microstructure of the final sugar-derived carbons at 1200 °C were characterized by FESEM and XRD, and the results are given in Figure 5 and Figure 6, respectively. It can be clearly observed from Figure 5 that the four carbons were almost similar in appearance. All of them were composed of irregular dense particles with diameters of several microns. According to the results of FTIR, the sugar in sugar–PAM was carbonized after heat treatment. Normally, the carbonization of sugar would cause formation of foaming due to the evaporation of volatile gases, but the dense structure showed that this did not happen in the heat treatment of sugar–PAM. That means the volatile component produced during the decomposition process would be trapped inside the three-dimensional network and cannot escape. This is the main reason that more carbon is retained.

Figure 6 shows the XRD patterns of the four as-prepared carbons. All the samples exhibited two board (002) and (100) diffraction peaks at about 24° and 43°, respectively. The (002) diffraction peak corresponds with graphene-like structure, and the (100) diffraction peak is related to the formation of disordered carbon material [42]. According to the Bragg equation, the values of interlayer space (d_002_) of four samples were approximate to 0.3660 nm, 0.3709 nm, 0.3737 nm and 0.3723 nm, respectively. All of them were far greater than the d_002_ value of graphite crystal (0.3354 nm), implying that the as-prepared sugar-derived carbons possessed a low degree of graphitization when carbonized at 1200 °C.

**Figure 7:**
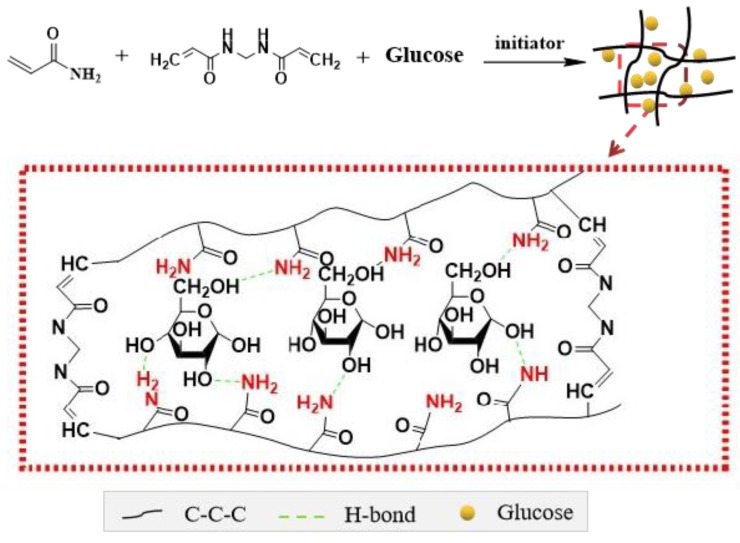
Structural diagram of the Glu–PAM hydrogel.

Overall, these results indicate that there were noncovalent rather than chemical cross-linking interactions between sugar and PAM, such as hydrogen bonds and intermolecular force. These results provide important insights into the structure of sugar–PAM, as shown in Figure 7 (take Glu–PAM, for example). When acrylamide polymerization was initiated in sugar aqueous solution, the formed PAM hydrogel with a three-dimensional network structure can absorb sugar solution due to the fact that PAM hydrogel acted as a hydrophilic polymer. The sugar solution was consequently separated into some small nanocapsules. Each nanocapsule might contain surrounding PAM walls and inner sugar molecules cross-linked with the walls by hydrogen bonds. It is this structure that prevents the normal foaming phenomenon of sugar at elevated temperature. The interchain hydrogen bonds between PAM and sugar played an important role in rapid self-recovery and energy dissipation. They can consolidate the whole hydrogel system to inhibit the expansion of sugar during thermal treatment. Nonetheless, sugar remained the main part to carbonize as it constitutes the major proportion of sugar–PAM. The whole carbonization of sugar–PAM is a general process of dehydration, condensation and aromatization.

## 4. Conclusions

In this work, four kinds of sugars (glucose, fructose, maltose, and sucrose) were selected as carbon precursors, and dense carbon products were formed using hydrogel carbonization. The PAM absorbed the sugar solution to form a hydrogel with a three-dimensional network structure in which existed a huge three-dimensional hydrogen bond network. The bonds not only consolidated the hydrogel system but also provided self-generated pressure to inhibit the foaming of sugar during heat treatment. Caramelization reaction rather than Maillard reaction occurred when sugar–PAM was carbonized. The main process during the carbonization of the four sugar–PAM hydrogels was similar, including degradation of intramolecular dehydration, condensation of intermolecular dehydration, and aromatization into rings. The four sugar-derived carbons had the low graphitization degree, in which glucose-derived carbon had the smallest value of d_002_.

## Figures and Tables

**Table 1:** The peak temperature of four sugar–PAM hydrogels in DTG curves.

Sugar–PAM	Peak 1 (°C)	Peak 2 (°C)	Peak 3 (°C)
Glu–PAM	208.9	288.1	
Fru–PAM	242.1		
Mal–PAM	139.4	242.9	298.5
Suc–PAM	229.1		

## Data Availability

Not applicable.
